# Transcriptome Analysis of Different Tissues Reveals Key Genes Associated With Galanthamine Biosynthesis in *Lycoris longituba*

**DOI:** 10.3389/fpls.2020.519752

**Published:** 2020-09-16

**Authors:** Qingzhu Li, Junxu Xu, Liuyan Yang, Xiaohui Zhou, Youming Cai, Yongchun Zhang

**Affiliations:** Forestry and Pomology Research Institute, Shanghai Academy of Agricultural Sciences, Shanghai Key Laboratory of Protected Horticultural Technology, Shanghai, China

**Keywords:** *Lycoris longituba*, galanthamine biosynthesis, transcriptomics, O-methyltransferase, functional characterization

## Abstract

*L* *ycoris longituba* is a traditional medicinal plant containing the bioactive compound galanthamine (Gal), a type of Amaryllidaceae alkaloid and can be used to treat Alzheimer’s disease. However, research on its genome or transcriptome and associated genes in the biosynthetic pathway is incomplete. In this study, we estimated the nuclear genome size of this species to be 29.33 Gb by flow cytometry. Then, RNA sequencing of the leaves, roots, and bulbs of *L. longituba* was carried out. After *de novo* assembly, 474,589 all-transcripts and 333,440 all-unigenes were finally generated. In addition, the differentially expressed genes (DEGs) were identified, and genes involved in the galanthamine metabolic pathway encoding tyrosine decarboxylase (TYDC), phenylalanine ammonia-lyase (PAL), cinnamate 4-hydroxylase (C4H), p-coumarate 3-hydroxylase (C3H), norbelladine synthase (NBS), norbelladine 4’-O-methyltransferase (OMT), and noroxomaritidine synthase (CYP96T1) were detected and validated by real-time quantitative PCR analysis. One candidate gene, *Lycoris longituba* O-Methyltransferase (*LlOMT*), was identified in the proposed galanthamine biosynthetic pathway. Sequence analysis showed that LlOMT is a class I OMT. LlOMT is localized in the cytoplasm, and biochemical analysis indicated that the recombinant LlOMT catalyzes norbelladine to generate 4′-O-methylnorbelladine. The protoplast transformation result showed that the overexpression of LlOMT could increase the Gal content. Our results indicate that LlOMT may play a role in galanthamine biosynthesis in *L. longituba*. This work provides a useful resource for the metabolic engineering of Amaryllidaceae alkaloids.

## Introduction

*Lycoris longituba* is a traditional medicinal plant of the Amaryllidaceae family and is endemic to the eastern temperate regions of China. Like other species in the *Lycoris* genus, the dried bulbs of *L. longituba* are used as a Chinese medicinal herb to treat carbuncle, sore throat, and edema (Wu et al., 2008). It has been reported that *L. longituba* contains an abundance of Amaryllidaceae alkaloids and is a valuable medicinal plant source (Guo et al., 2014; Li Q. Z. et al., 2018). To date, more than 500 alkaloids with different structures have been isolated (Jin, 2013; Singh and Desgagné-Penix, 2014). Phytochemical studies show that these alkaloids have many pharmacological functions. For example, narciclasine has antitumor activity (Kornienko and Evidente, 2008), lycorine can block the cell cycle and induce apoptosis of the HL-60 cancer cell line (Liu et al., 2004), and haemanthamine has anticancer activity (Pellegrino et al., 2018). Galanthamine (Gal) is a unique isoquinoline alkaloid. As an acetylcholinesterase inhibitor, it can affect the brain nicotine receptor and inhibit the activity of acetylcholinesterase (Barnes et al., 2000; Wang et al., 2010). Gal is one of the drugs used for the treatment of Alzheimer’s disease, and its efficacy and safety in the treatment of Alzheimer’s disease have been confirmed by a clinical trial (Repantis et al., 2010).

In contrast to the extensive literature on the pharmaceutical effects of Gal, information on its biosynthesis pathway is incomplete. The proposed Gal biosynthesis pathway in *Narcissus* showed that the core intermediate norbelladine was synthesized from 3,4-dihydroxybenzaldehyde and tyramine, and then transformed into 4’-O-methylnorbelladine under the catalysis of norbelladine 4’-O-methyltransferase (OMT). 4’-O-Methylnorbelladine is oxidized to N-demethylnarwedine, following which it is reduced to N-demethylgalanthamine, and then finally N-demethylgalanthamine is methylated to galanthamine (Kilgore et al., 2014). The *NpN4OMT* and *CYP96T1* genes have been reported to be involved in the Gal biosynthesis pathway in *Narcissus* (Kilgore et al., 2014; Kilgore et al., 2016). In *Lycoris*, only a few studies on *L. radiata* and *L. aurea* have reported genes, including *LrPAL*, *LrC4H*, and *LaOMT1*, that may be involved in the Gal pathway (Li et al., 2018; Sun et al., 2018).

In this study, the genome size of *L. longituba* was determined by flow cytometry. RNA from the roots, leaves, and bulbs was extracted to construct cDNA libraries for transcriptome analysis. Our aim was to explore the transcriptome of the non-model plant *L. longituba* and to study the biosynthesis of the Amaryllidaceae alkaloid Gal. One of the key genes in the pathway name LlOMT1 was characterized, and six putative genes involved in the galanthamine biosynthetic pathway of *L. longituba* were discovered. This work will provide a useful resource for the metabolic engineering of Amaryllidaceae alkaloids.

## Materials and Methods

### Plant Materials

**Figure 1:**
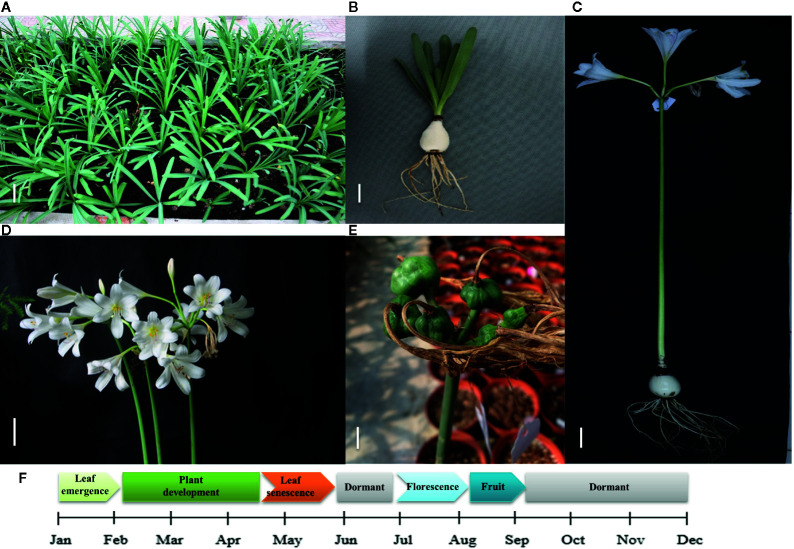
Growth cycle of *L. longituba* in this study. **(A)** The plant growth of *L. longituba* at the plant development stage. **(B)** The leaf, bulb, and root of *L. longituba* at the plant development stage. **(C)** The whole plant of *L. longituba* at the flowering stage. **(D)** Inflorescence of *L. longituba* at the flowering stage. **(E)** Fruit of *L. longituba*. **(F)** Growth period of *L. longituba* during the growth cycle.

*Lycoris longituba* plant materials were collected from the resource nursery at the Shanghai Academy of Agricultural Sciences in China (31.23°N, 121.10°E) and identified by Prof. Zheng Yuhong (Institute of Botany, Chinese Academy of Sciences, Jiangsu Province). The material consistency was maintained through micropropagation. Voucher specimens of *L. longituba* (0653969) were deposited at the herbarium of the Institute of Botany, Chinese Academy of Sciences. Total RNA was extracted from the leaves (L), roots (R), and bulbs (B) of actively growing three-year-old samples. Experiments were conducted in three independent biological replicates (**Figure 1**).

### Nuclear DNA Content Determination

Seedlings of *Hordeum vulgare* L. cv. Morex were supplied by Longhua Zhou (Shanghai Academy of Agriculture Sciences, Shanghai, China), which was used as standard [≈5.1 Gb (Mayer et al., 2012)]. The young leaves were collected from *Hordeum vulgare* L. and *L. longituba* seedlings. The leaves were homogenized in 2 ml of homogenization buffer [45 mM MgCl_2_; 30 mM sodium citrate; 20 mM 3-morpholinopropanesulfonic acid; 0.1% (*w/v*) TritonX-100; pH 7.0] and then filtered with a 200-mesh nylon net (Galbraith et al., 1983). Centrifugation of the filtrate was conducted at 1500 rpm for 5 min to obtain the nuclear fraction. The obtained fraction was then stained with propidium iodide (PI; 50 µg/ml), incubated at 4°C for 20 min, and again filtered through a 500-mesh nylon net. The resulting nuclear suspensions were sorted *via* flow cytometry. Experiments were conducted in three independent biological replicates.

### RNA Extraction and cDNA Library Construction

mRNA extraction, cDNA library construction, and library sequencing were all performed by Shanghai Personal Biotechnology Co., Ltd (Shanghai, China). Total RNA was extracted from plant materials using the TRlzol reagent from nine samples (three tissues with three independent biological replicates), followed by digestion with DNase I and enrichment with Oligo (dT) magnetic beads. The RNAs were then fragmented with a fragmentation buffer. First-strand cDNA was synthesized from the RNA pools by using random primers and conversion to double-stranded cDNA with RNase H and DNA polymerase I. A QIAquick PCR Extraction Kit (Qiagen, Valencia, CA, USA) was then used to purify fragments of appropriate length. The termini of the DNA fragments were end-repaired and ligated with sequencing adapters. AMPure XP beads (Beckman Coulter, Shanghai, China) were used to remove fragments that were incorrectly synthesized. Polymerase chain reaction (PCR) was then used to construct fragment libraries that were quantified using the PicoGreen kit (Quantifluor™-ST fluorometer E6090, Promega, CA, USA) and fluorospectrophotometry (Quant-iT PicoGreen dsDNA Assay Kit; Invitrogen, P7589). The cDNA pools were then quantified with an Agilent 2100 Bioanalyzer (Agilent Technologies, Santa Clara, CA, USA; Agilent High Sensitivity DNA Kit, Agilent, 5067–4626). Synthesized cDNA libraries were normalized to 2-nM concentrations. The nine sample libraries were then gradually diluted, quantified to 4–5 pM, and sequenced on the Illumina HiSeq X Ten platform.

### Sequencing, *de novo* Assembly, and Transcript Annotation

A total of approximately 20 Gbp of sequence data were generated for each sample (three tissues with three independent biological replicates). The raw data were quality filtered to remove reads with adapters or those that contained more than five ambiguous nucleotides (“N”). Low-quality reads were then removed before subsequent analysis. The Trinity software program (Grabherr et al., 2011) was used to assemble the remaining high-quality “clean” reads. Annotations were conducted *via* Basic Local Alignment Search Tool (BLAST) searches of sequences against the Kyoto Encyclopedia of Genes and Genomes (KEGG) (Kanehisa and Goto, 2000) and National Centre for Biotechnology Information (NCBI) non-redundant (nr) protein databases and Swiss-Prot database in addition to the NCBI non-redundant nucleotide (nt) database. An *E*-value cutoff of 10^−5^ was used to assign annotations based on matches in the databases.

### Differential Gene Expression Analysis and Functional Enrichment

Clean reads were aligned to unigenes, and the reads per kilobase of exon per million mapped reads (RPKM) values were calculated for the unigenes from the L, R, and B samples. Differential expression of the unigenes was then calculated using the DEGseq software package, with fold-change thresholds set *a priori* to *P* ≤ 0.05 and a log2 (fold change) > 1. The Gene Ontology (GO), KEGG, and eggNOR databases (Mortazavi et al., 2008; Wang et al., 2009; Kanehisa et al., 2012) were then used to further analyze the differentially expressed genes (DEGs). Analysis was conducted in three independent biological replicates.

### Real-Time Quantitative PCR (RT-qPCR) Validation of Gene Expression

Total RNA was extracted from the L, R, and B samples of *L. longituba* using the TRIzol reagent (Invitrogen) according to the manufacturer’s instructions. RNA samples were then treated with DNAase I (Promega, USA) and reverse-transcribed using the M-MLV reverse transcriptase (Promega, USA) to generate cDNA for gene expression analysis. RT-qPCR assays were conducted using the SYBR Green Mix (Applied Biosystems) and amplification in a StepOne Plus real-time PCR system with the following conditions: pre-incubation (95°C for 5 min), followed by 40 cycles of 95°C for 15 s and 60°C for 30 s. The CT value comparative method (Schmittgen and Livak, 2008) was used to determine expression level fold changes for the target genes. Gene specific primers were designed on the basis of the cDNA sequences for real-time PCR amplification of corresponding genes according to the instructions of real-time PCR system. The Actin gene from *L. longituba* was used as an internal reference. Primer sequence information used in RT-qPCR assays is provided in **Supplementary Table 1**. Experiments were conducted in three independent biological replicates.

### Alkaloid Extraction and Quantification

GAL was determined according to a previous method (Li W. et al., 2018). Approximately 0.2 g plant tissue was freeze-dried and extracted in 2 ml of 70% high-performance liquid chromatography (HPLC)–grade ethanol by sonication. After centrifugation at 12,000 rpm for 10 min, the supernatants were vacuum-dried and re-dissolved in 1 ml of 0.1% formic acid-acetonitrile (V/V = 95/5) for LC tandem mass-spectrometry (MS/MS) analysis. Gal was purchased from TCI Development Co., Ltd (Shanghai, China). HPLC-grade solvents (methanol, formic acid, water, ethanol, acetonitrile) were obtained from Thermo Fisher Scientific (USA). Analysis of the GAL was performed with a Nexera ultra(U)HPLC LC-30A (Shimadzu) equipped with a Waters ACQUITY UPLC BEH C18 column (150 mm×2.1 mm, 1.7 μm). Separation was conducted using 0.1% formic acid (v/v) (A) and acetonitrile (B) with a 6-min linear gradient of 5%–60% B at a flow rate of 0.2 ml/min. The LC elution was monitored using an API-5500-QTRAP mass spectrometer (AB SCIEX) operating in positive detection mode. The transition reactions m/z 288→231 were used for the quantification of GAL. Experiments were conducted in three independent biological replicates.

### Cloning and Sequence Analysis of the LlOMT Gene

LlOMT coding sequences were amplified by PCR with gene-specific primers (**Supplementary Table 1**). The amino acid sequences of the different OMTs were then aligned using Clustal Omega (http://www.clustal.org/omega/). Phylogenetic analysis was conducted with 29 OMT proteins using MEGA X 10.1 (https://www.megasoftware.net/) with the neighbor-joining method, and node support was determined using 1,000 bootstrap replicates.

### Localization Analysis of LlOMT

The LlOMT coding region was cloned in-frame with the green fluorescent protein (GFP) gene in the pBWA(V)HS-35S-NOS vector. *Agrobacterium tumefaciens* (strain GV3101) cultures harboring this construct were adjusted to OD_600_ = 0.8 in MES buffer (10 mM MES, pH 5.5, and 10 mM MgSO_4_) and then infiltrated in 6-week-old *Nicotiana benthamiana* leaves, as described previously (Liu et al., 2016). After 3 days, the fluorescent signals in the inoculated leaves were examinedwith confocal microscopy (LSM710, Carl Zeiss Microscopy). Experiments were conducted in three independent biological replicates.

### Enzyme Activity Assay and Kinetics Parameters of LlOMT

The LlOMT coding sequence with a His tag was cloned in-frame in the pET–28a vector (Novagen) and transformed into *Escherichia coli* BL21 (DE3) cells to express the recombinant protein. The His-tag fusion protein was then purified using Ni-NTA agarose (Qiagen) following the manufacturer’s instructions. LlOMT enzyme activity assays were conducted according to Kilgore’s method (2014). The 100-μl (final volume) solution contained 10 μg recombinant protein, 200 μM AdoMet, and 100 μM norbelladine in 30 mM potassium phosphate buffer (pH 8.0). Reactions were incubated at 30°C for 2 h and terminated by adjusting the pH to 9.5 with two volumes of sodium bicarbonate, followed by two extractions in ethyl acetate. The extracts were vacuum-dried and re-dissolved in 100 μl of 50% methanol for measurement in LC-MS/MS analysis. To determine the kinetics parameters of LlOMT-catalyzed O-methylation, the reactions were performed in 100 μl solution contained 10 μM recombinant protein, 200 μM AdoMet, and 50 μM-2 mM norbelladine in 30 mM potassium phosphate buffer (pH 8.0), reactions were incubated at 30°C for 2 h. Reaction products were measured by a Nexera UHPLC LC-30A system (Shimadzu) equipped with a Waters ACQUITY UPLC BEH C18 (150 mm × 2.1 mm, 1.7 μm). Separation was carried out using 0.1% formic acid (v/v) (A) and methanol (B) with a 6-min linear gradient of 5%–40% B at a flow rate of 0.4 ml/min. The LC elution was monitored using an API-4000-QTRAP mass spectrometer (AB SCIEX) operating in positive detection mode for norbelladine and 4’-O-methylnorbelladine. Identification was based on retention times and MS/MS spectra compared to authentic standards (Shanghai spectrum chemical Biotechnology Co., Ltd), The transition reactions *m/z* 260→138 and 274→137 were used for the quantification of norbelladine and 4’-O-methylnorbelladine. Experiments were conducted in three independent technical replicates.

### Protoplast Transformation

The LlOMT-GFP fusion constructs were transformed into *L. longituba* protoplasts using polyethylene glycol (PEG)-mediated transformation according to our patent (CN 201910010502.5). The GFP empty vector was used as a negative control. Experiments were conducted in three independent biological replicates. Generally, 1.5 g leaf of *L. longituba* seedlings was cut into small pieces and quickly transferred into 10 ml A buffer (1.5% cellulose R10 (Yakult), 0.75% macerozyme R10 (Yakult), 0.5M mannitol, 10mM CaCl_2_, 0.1% bovine serum albumin, 10mM MES pH = 5.7), following which the samples were incubated for 4 h at 28°C with gentle shaking. Afterward, the enzyme solution was filtered through a 50-µm cell strainer to collect the protoplasts, and the residue was combined with B buffer (154mM NaCl, 125mM CaCl_2_, 5 mM KCl, 2mM MES pH = 5.7) and filtered again. The flow-through was centrifuged at 150 g for 5 min and the pellet was washed twice with B buffer. The protoplasts were re-suspended in C buffer (0.5 M mannitol, 15mM MgCl_2_, 4mM MES pH = 5.7) and adjusted to 1 × 10^7^ cells per ml. Protoplasts were mixed with plasmids and D buffer [40% (W/V) polyethylene glycol, 0.1M CaCl_2_, 0.2M mannitol]. The mixture was incubated at 28°C for 15 min and then diluted with 1 ml B solution, followed by centrifugation at 150 g for 5 min. The protoplasts were re-suspended in 1 ml B buffer and incubated at 28°C for 12 h. After incubation, fluorescent signals in the transformed protoplasts were examined by confocal microscopy (LSM710, Carl Zeiss Microscopy).

## Results

### Genome Size Estimation

To determine the genome size of *L. longituba*, the young leaf nucleus of *L. longituba* was analyzed using flow cytometry with *H. vulgare* [≈5.1 Gb (Mayer et al., 2012; Mascher et al., 2017)] as an internal standard. The genome size of *L. longituba* [2n = 16 (Ke et al., 1998)] was estimated to be 29.33 ± 0.08 Gb (**Table 1** and **Supplementary Figure 1**). This result is similar with a previous estimation of about 30.675 Gb (Jiang et al., 2017) and will provide a reference for the genomic research of *L. longituba* and for the evaluation of sequencing depth.

**Table 1:** Flow cytometry determination of the nuclear genome size of *L. longituba* (*N* = 3).

Lycoris longituba. peak	Hordeum vulgare peak	Peak ratio (Lycoris longituba/Hordeum vulgare)	Lycoris longituba genome size (Gb mean ± SD)
857	149	5.7517	29.3337
859	149	5.7651	29.4020
866	151	5.7351	29.2490
			29.33 ± 0.08

SD, standard deviation.

### Illumina Sequencing and Reads Assembly

For a non-model species that lacks genome information, such as *L. longituba*, *de novo* transcriptome analysis can facilitate the discovery of novel genes and elucidate complex metabolic pathways. To generate a transcriptome database, nine total mRNA libraries were generated by Illumina sequencing, including three from *L. longituba* leaves, three from bulbs, and three from roots.

A total of approximately 133 million raw reads were recovered from the root libraries, 134 million from the leaves, and 139 million from the bulbs. After filtering out adapter sequences and low-quality reads, over 131, 132, and 137 million clean reads were recovered for the R, L, and B samples, respectively. The clean reads from each sample were assembled resulting in 360,593 (R), 320,299 (L), and 326,962 (B) transcripts. The average transcript size exceeded 750 bp for each tissue library, with an N50 of 1,133–1,195 bp. A total of 223,969 (R), 185,467 (L), and 191,716 (B) unigenes were identified in the datasets. After conducting long-sequence clustering of the nine samples, 474,589 all-transcripts and 333,440 all-unigenes were ultimately generated. These datasets had mean lengths of 647 and 521 bp and N50s of 981 and 644 bp, respectively. The length distributions of the transcripts and unigenes are shown in **Supplementary Figure 2**, while a summary of the sequencing and assembly results is provided in **Table 2**. All raw data have been deposited in the NCBI Sequence Reads Archive (SRA) with the accession number PRJNA590043.

**Table 2:** Summary of the Illumina sequencing and reads assembly for *L. longituba* (*N* = 3).

	Roots (R)	Leaves (L)	Bulbs (B)	Total
No. of raw reads	1.33×10^8^	1.35×10^8^	1.39×10^8^	4.07×10^8^
Length of raw reads (bp)	2.00×10^10^	2.02×10^10^	2.09×10^10^	6.12×10^10^
No. of clean reads	1.31×10^8^	1.33×10^8^	1.37×10^8^	4.02×10^8^
Length of clean reads (bp)	1.93×10^10^	1.97×10^10^	2.03×10^10^	5.93×10^10^
No. of transcripts	3.60×10^5^	3.20×10^5^	3.27×10^5^	4.74×10^5^
Length of transcripts (bp)	2.74×10^8^	2.61×10^8^	2.63×10^8^	3.07×10^8^
Average length of transcripts (bp)	7.59×10^2^	8.17×10^2^	8.05×10^2^	6.48×10^2^
N50 of transcripts (bp)	1.13×10^3^	1.19×10^3^	1.19×10^3^	9.81×10^2^
N90 of transcripts (bp)	3.22×10^2^	3.57×10^2^	3.47×10^2^	2.67×10^2^
No. of unigenes	2.24×10^5^	1.85×10^5^	1.92×10^5^	3.33×10^5^
Length of unigenes (bp)	1.42×10^8^	1.30×10^8^	1.31×10^8^	1.74×10^8^
Average length of unigenes (bp)	6.32×10^2^	7.01×10^2^	6.85×10^2^	5.21×10^2^
N50 of unigenes (bp)	8.49×10^2^	9.55×10^2^	9.47×10^2^	6.44×10^2^
N90 of unigenes (bp)	2.77×10^2^	3.12×10^2^	2.99×10^2^	2.43×10^2^

### Functional Annotation

**Figure 2:**
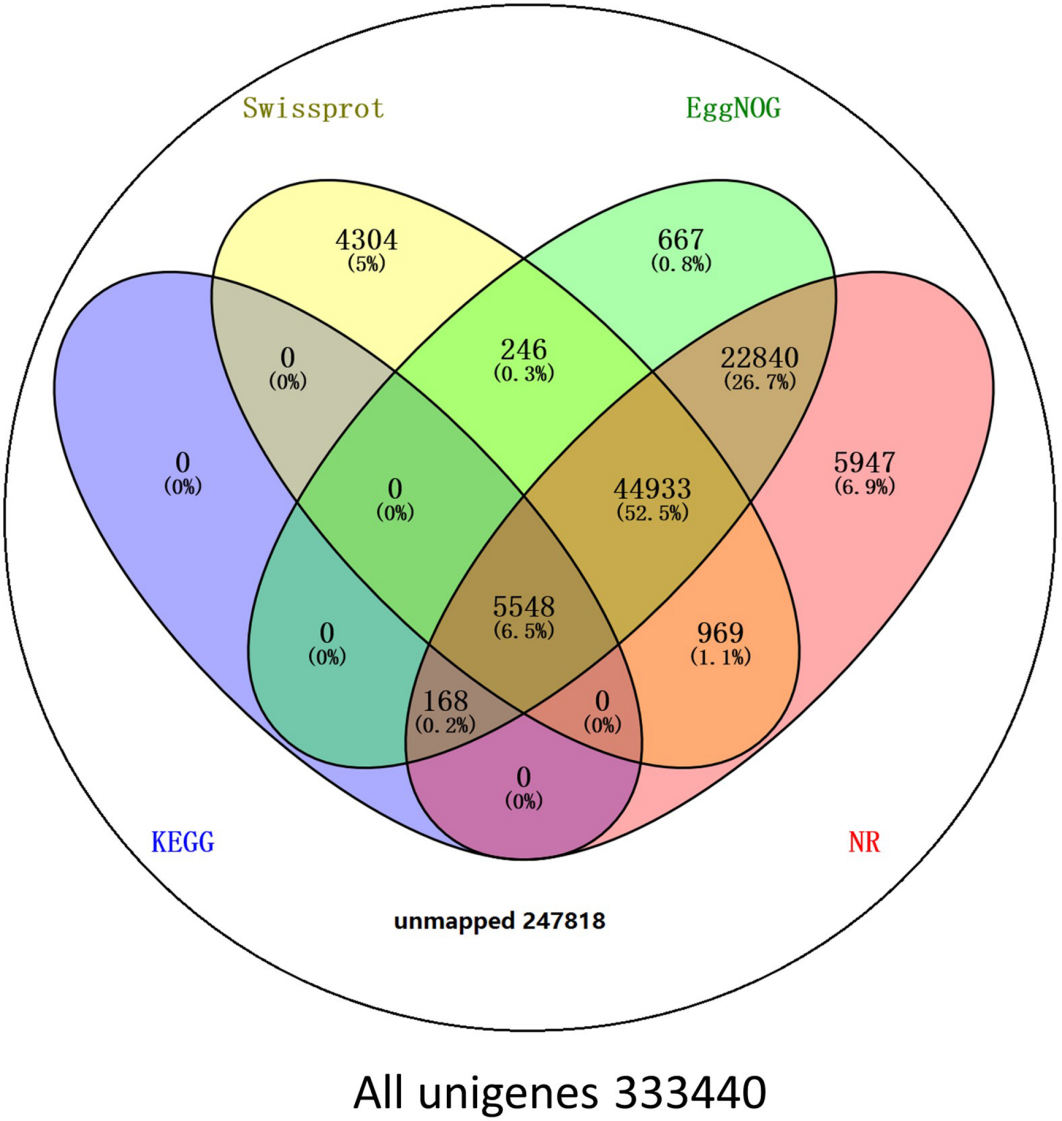
Number of unigenes blasted to Nr, eggNOG, Swiss-Prot, and KEGG (E < 0.00001). KEGG, Kyoto encyclopedia of genes and genomes (*N* = 3).

BLAST comparisons of the all-unigenes datasets against non-redundant (nr), eggNOG, Swiss-Prot, and KEGG databases indicated that 85,622 (25.68%) of the unigenes possessed homologous sequences in at least one of the above databases. A total of 80,405 (24.11%), 74,402 (22.31%), 56,000 (16.79%), and 5716 (1.71%) unigenes had homologs in the nr, eggNOG, Swiss-Prot, and KEGG databases, respectively. A total of 2917 (0.87%) unigenes exhibited homologs in all three databases, while 247,817 (74.32%) unigenes did not have representative homologs in the databases (**Figure 2**). These transcripts may thus represent novel proteins, long non-coding RNAs in the *L. longituba* genome, or they could be derived from less conserved 3′- or 5′-untranslated regions of genes.

We obtained the species homology distribution of the *L. longituba* unigenes *via* nr annotations. Comparisons against 1,172 plant species with homologous mRNA sequences to those of *L. longituba* indicated that the annotated unigenes were most similar to those of *Elaeis guineensis* (12.91%), *Phoenix dactylifera* (12.73%), and *Musa acuminata* subsp. *malaccensis* (4.83%) (**Supplementary Figure 3**).

Transcription factors (TFs) comprise diverse gene families that play key regulatory roles in plant growth and development by controlling the expression of genes through specific binding to *cis*-regulatory elements that are present in the promoter regions of target genes. Many TF families are known to regulate secondary metabolite biosynthesis in plants including the MYB, MYB-related, WRKY, and bHLH families (Park et al., 2008; Hichri et al., 2010; Xie et al., 2016; Yin et al., 2017; Yu et al., 2018). Our analysis of the *L. longituba* transcriptome data revealed that 38347 unigenes (11.50%) encode putative TFs that can be classified into 58 TF families (**Supplementary Table 2**). Members of the bHLH TF family were the most abundant (3115, 8.12%) followed by ERF (2882, 7.51%), NAC (2466, 6.43%), TCP (2312, 6.03%), and MYB-related (2250, 5.87%) (**Supplementary Figure 4** and **Supplementary Table 2**). The identification of these abundant TFs combined with their expression profiles in individual tissues provides a rich resource for future characterization of specific TFs in various biochemical pathways of *L. longituba*.

To further evaluate the primary biological functions represented within the transcriptomes, GO, eggNOG, and KEGG pathway analyses were performed. GO mapping provides a description of gene products based on their associated biological processes (BPs), cellular components (CCs), and molecular functions (MFs) (Berardini et al., 2004) (**Supplementary Figure 5**). A total of 32,128 unigenes were categorized into 67 major functional groups. Metabolic process (GO:0008152), cell (GO:0005623), and binding (GO:0005488) were the most highly represented GO terms in the BP, CC, and MF transcriptomes, respectively (**Supplementary Table 3** and **Supplementary Figure 5**).

eggNOG mapping was also performed to further evaluate the function of the assembled unigenes. A total of 74,402 annotated unigenes were grouped into 26 eggNOG classification groups (**Supplementary Table 4** and **Supplementary Figure 6**). The three most abundant groups represented in the transcriptomes [excluding groups R (general function prediction only) and S (function unknown)] were group L (replication, recombination, and repair), group O (post-translational modification, protein turnover, and chaperones), and group T (signal transduction mechanisms) (**Supplementary Table 4**).

A total of 5,716 unigenes were mapped to 35 major KEGG pathways (**Supplementary Tables 3**, **5**). The pathways with the highest unigene representations were translation (516 unigenes), carbohydrate metabolism (403 unigenes), and signal transduction (398 unigenes).

### Differential Expression Analysis

**Figure 3:**
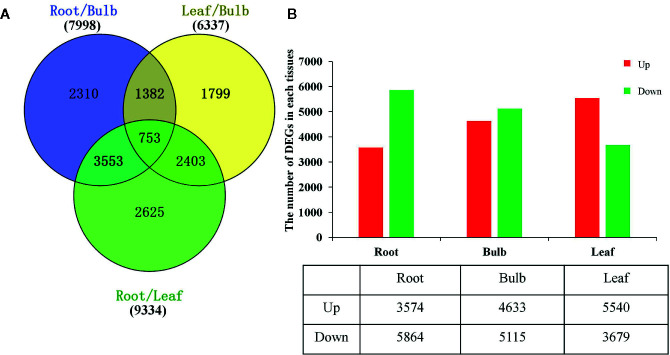
Differential expression analysis of *L. longituba* unigenes. **(A)** Venn diagram representing the number of DEGs in different pairwise comparisons. **(B)** The number of significantly (*P-*value ≤ 0.05 and at least two-fold change) Up- and Down- regulated unigenes in each tissue compared to all other tissues. DEGs, differentially expressed genes (*N* = 3).

The DEGs were identified based on the normalized RPKM value for each transcript in individual tissues (**Supplementary Figure 7** and **Supplementary Tables 6**, **7**). A total of 14,825 DEGs were detected in at least one pairwise comparison among the R/B, L/B, and R/L comparisons (**Figure 3A**). DEGs were further compared in each tissue relative to the other two tissues using thresholds of *P* < 0.05 and at least a two-fold expression change among different tissues. Specifically, DEGs were identified by comparing their RPKM values from one tissue against those of the other two tissues in order to compare an equivalent statistical parameter. A total of 3,574 unigenes were up-regulated in roots, while 5,864 unigenes were down-regulated relative to their expression in the other two tissues (**Figure 3B**). The bulbs had the largest number of DEGs (9748), with 4,633 up-regulated and 5,115 down-regulated unigenes. The leaves had the largest number of up-regulated unigenes, with 5,540 up-regulated and 3,679 down-regulated unigenes (**Figure 3B**).

Furthermore, we identified 324 DEGs that were assigned functions within 28 secondary metabolic pathways of KEGG (**Table 3**). Among these, 40 unigenes encoded key enzymes involved in terpenoid biosynthesis pathways, including the synthesis of the terpenoid backbone (28 unigenes), monoterpenoids (two unigenes), diterpenoids (seven unigenes), and sesquiterpenoids and triterpenoids (three unigenes). Further, 29 DEG unigenes were identified in the ﬂavonoid biosynthesis pathway, including biosynthetic pathways for phenylpropanoid (17 unigenes), ﬂavonoids (11 unigenes), and ﬂavones and ﬂavonols (one unigene). Thirty unigenes were associated with porphyrin and chlorophyll metabolism pathways, while only seven unigenes were associated with isoquinoline alkaloid biosynthesis. The identification and future characterization of these unigenes that are involved in various metabolic pathways will help to better understand their functions in the biosynthesis of active compounds by *L. longituba* plants.

**Table 3:** Secondary metabolic pathways and their related number of DEGs in the two samples as compared with bulbs (*N* = 3).

Pathway ID	Pathways	Unigene number	Functional categories
ko00100	Steroid biosynthesis	16	17
ko00130	Ubiquinone and other terpenoid-quinone biosynthesis	20	29
ko00230	Purine metabolism	89	122
ko00232	Caffeine metabolism	2	2
ko00362	Benzoate degradation	2	2
ko00400	Phenylalanine, tyrosine and tryptophan biosynthesis	21	31
ko00625	Chloroalkane and chloroalkene degradation	2	4
ko00627	Aminobenzoate degradation	3	4
ko00760	Nicotinate and nicotinamide metabolism	12	14
ko00860	Porphyrin and chlorophyll metabolism	30	36
ko00900	Terpenoid backbone biosynthesis	28	32
ko00901	Indole alkaloid biosynthesis	1	1
ko00902	Monoterpenoid biosynthesis	2	2
ko00903	Limonene and pinene degradation	1	2
ko00904	Diterpenoid biosynthesis	7	13
ko00905	Brassinosteroid biosynthesis	7	9
ko00906	Carotenoid biosynthesis	19	24
ko00908	Zeatin biosynthesis	5	13
ko00909	Sesquiterpenoid and triterpenoid biosynthesis	3	3
ko00940	Phenylpropanoid biosynthesis	17	77
ko00941	Flavonoid biosynthesis	11	19
ko00942	Anthocyanin biosynthesis	1	1
ko00944	Flavone and flavonol biosynthesis	1	1
ko00945	Stilbenoid, diarylheptanoid and gingerol biosynthesis	5	10
ko00950	Isoquinoline alkaloid biosynthesis	7	11
ko00960	Tropane, piperidine and pyridine alkaloid biosynthesis	7	13
ko00965	Betalain biosynthesis	2	2
ko01220	Degradation of aromatic compounds	3	4

DEGs, differentially expressed genes.

### Gene Expression Analysis of the DEGs Involved in Gal Biosynthesis in the Different Tissues of *L. longituba*

**Figure 4:**
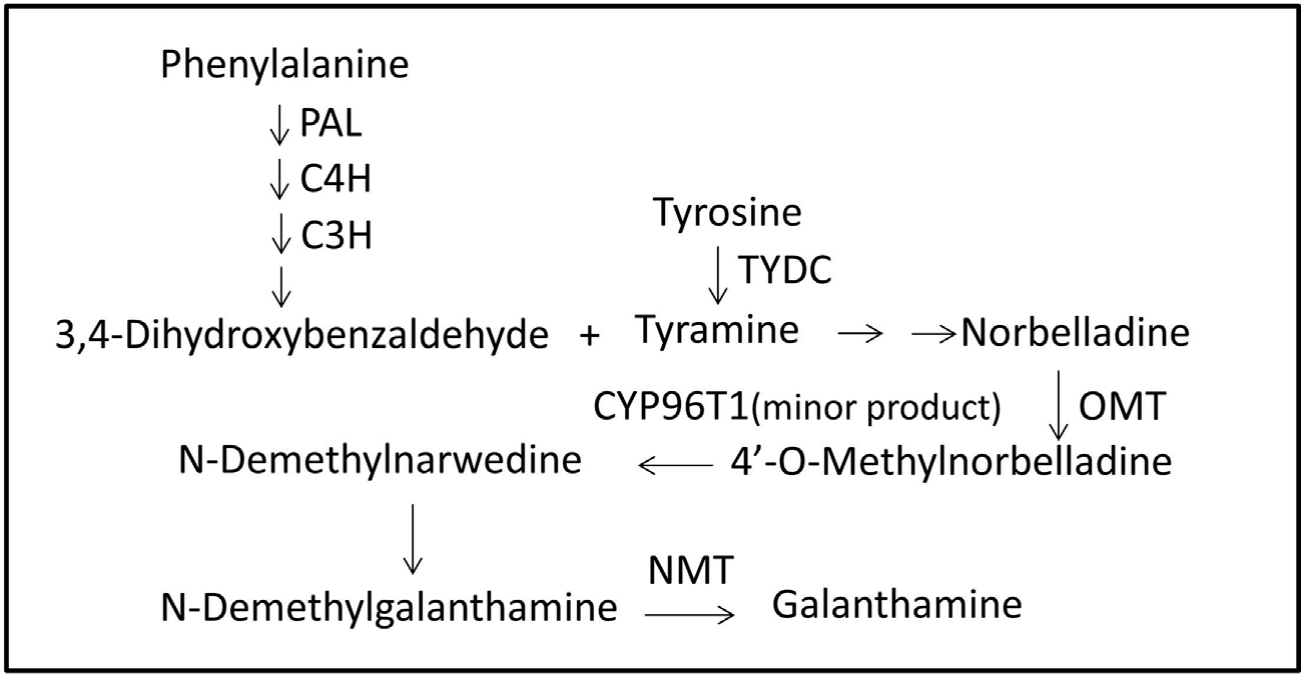
Proposed Gal biosynthesis pathway in *L. longituba.* Norbelladine is synthesized from 3,4-dihydroxybenzaldehyde and tyramine, and then transformed into 4’-O-methylnorbelladine under the catalysis of OMT. 4’-O-methylnorbelladine is oxidized to N-demethylnarwedine, and then, it is reduced to N-demethylgalanthamine, and finally N-demethylgalanthamine is methylated to galanthamine. C3H, p-coumarate 3-hydroxylase; C4H, Cinnamate 4-hydroxylase; CYP96T1, noroxomaritidine synthase; Gal, galanthamine; NBS, norbelladine synthase; NMT, N-methyltransferase; OMT, norbelladine 4’-O-methyltransferase; PAL, phenylalanine ammonia; TYDC, tyrosine decarboxylase.

**Figure 5:**
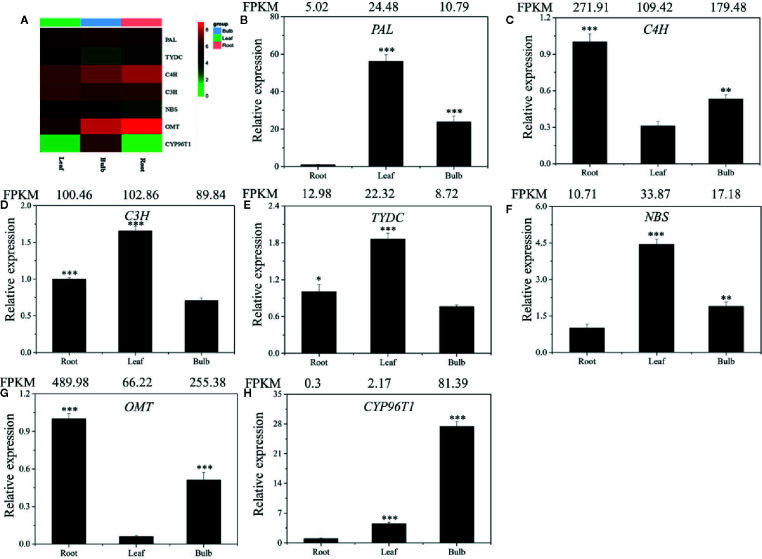
Expression analysis of the DEGs involved in Gal biosynthesis in the different tissues of *L. longituba*. Heatmap **(A)** and RT-qPCR analysis of *PAL* **(B)**, *C4H* **(C)**, *C3H* **(D)**, *TYDC* **(E)**, *NBS* **(F)**, *OMT* **(G)**, and *CYP96T1* **(H)**. The genes selected for validation were the RNA-Seq data. The bar chart shows the relative expression levels as measured by RT-qPCR values, while the top vertical axis shows the relative RPKM values. Experiments were conducted in triplicate. Error values indicate the standard deviation (SD). C3H, p-coumarate 3-hydroxylase; C4H, Cinnamate 4-hydroxylase; CYP96T1, noroxomaritidine synthase; DEGs, differentially expressed genes; NBS, norbelladine synthase; OMT, norbelladine 4’-O-methyltransferase; PAL, phenylalanine ammonia; RPKM, Reads Per Kilobase per Million mapped reads; RT-qPCR, real-time quantitative polymerase chain reaction; TYDC, tyrosine decarboxylase (*N* = 3, *P < 0.05, **P < 0.01, ***P < 0.001 by Student’s *t*-test).

Local BLASTx analyses were conducted to identify gene transcripts encoding enzymes that are putatively involved in Gal biosynthesis (**Figure 4**). Several transcript isoforms of orthologous genes were identified from the precursor pathway leading to norbelladine. For example, the PAL gene has been cloned and characterized from *L. radiata*, from which LrPAL3 was identified (Li et al., 2018). BLASTx searches for the PAL3 gene in the transcriptome led to the identification of one transcript with 93.1% similarity to the PAL3 sequence, with an E-value of zero. Thus, we identified this as LlPAL (**Table 4**). Similarly, one gene transcript showed 91.8% similarity to the *Narcissus aff. pseudonarcissus* TYDC, with an E-value of zero (Singh and Desgagné-Penix, 2017). Thus, we were able to find seven transcripts with E-values corresponding to genes encoding enzymes involved in Gal biosynthesis (Kilgore et al., 2014; Kilgore et al., 2016; Singh and Desgagné-Penix, 2017; Li et al., 2018; Sun et al., 2018). In the central Gal biosynthetic pathway, sequence reads corresponding to OMT, C4H, and C3H were more abundant than those operating in precursor pathways including PAL, TYDC, and others (**Figure 5A**). This result was confirmed by FPKM digital expression comparisons and through RT-qPCR analysis (**Figures 5B–H**).

**Table 4:** Summary of the putative biosynthetic genes involved in Gal biosynthesis in different tissues of *L. longituba* (*N *= 3).

Name	Length (bp)	Annotation	Species	E-value	Similarity (%)	Accession No.
PAL	2127	Phenylalanine ammonia-lyase	*Lycoris radiata*	0	93.1	c147928_g2
TYDC	504	Tyrosine decarboxylase	*Narcissus aff. pseudonarcissus*	0	91.8	c141141_g1
C4H	1509	Cinammate 4-hydroxylase	*Lycoris radiata*	0	93.6	c145306_g3
C3H	1461	p-coumarate 3-hydroxylase	*Narcissus pseudonarcissus*	0	94.7	c144663_g1
NBS	474	norbelladine synthase	*Narcissus aff. pseudonarciss*	5.4E-169	88.22	c142088_g2
OMT	717	Norbelladine 4’-O-methyltransferase	*Narcissus aff. pseudonarcissus*	4.0E-161	91.2	c141146_g2
CYP96T1	1536	Noroxomaritidine synthase	*Narcissus aff. pseudonarcissus*	0	90.3	c146899_g2

C3H, p-coumarate 3-hydroxylase; C4H, cinnamate 4-hydroxylase; CYP96T1, noroxomaritidine synthase; NBS, norbelladine synthase; OMT, norbelladine 4’-O-methyltransferase; PAL, phenylalanine ammonia-lyase; TYDC, tyrosine decarboxylase.

To validate the gene expression profiles across tissues, we performed RT-qPCR analysis. The expression profiles of the seven proposed Gal biosynthetic genes were analyzed in the different plant tissues (B, R, and L) (**Figures 5B–H**). *C4H* and *OMT* showed high expression in R compared to the other tissues. *PAL*, *C3H*, *TYDC*, and *NBS* were relatively highly expressed in L. *CYP96T1* was highly expressed in B compared to the other tissues, which did not correlate with the expression of *OMT* (**Figure 5**). The RT-qPCR results matched the FPKM results well. Taken together, these results indicated that the different genes are expressed differently across the tissues.

### Cloning and Sequence Analysis of LlOMT

The *LlOMT* gene was obtained from transcriptome sequencing of *L. longituba* (GenBank IDMK883815). The open reading frame (ORF) of the *LlOMT* gene was 720 bp, encoding a protein of 239 amino acids with a molecular weight of approximately 29.17 kDa and an isoelectric point (pI) of 4.87 (**Supplementary Figure 8**). LlOMT is a conserved homolog of NpN4OMT and LaOMT1 with 91.2% and 98.7% amino acid sequence identity (**Supplementary Figure 8A**) (Kilgore et al., 2014; Sun et al., 2018). To infer the phylogeny of LlOMT, phylogenetic analysis was conducted with LlOMT homologs from taxonomically diverse plant species using neighbor-joining phylogenetic reconstruction methods. As shown in **Supplementary Figure 8B**, two distinct clusters of OMT homologs were identified. Like NpN4OMT, LlOMT belongs to the class I OMT group and harbors the same types of functional domains (**Supplementary Figure 8**).

### Subcellular Localization Analysis of LlOMT

**Figure 6:**
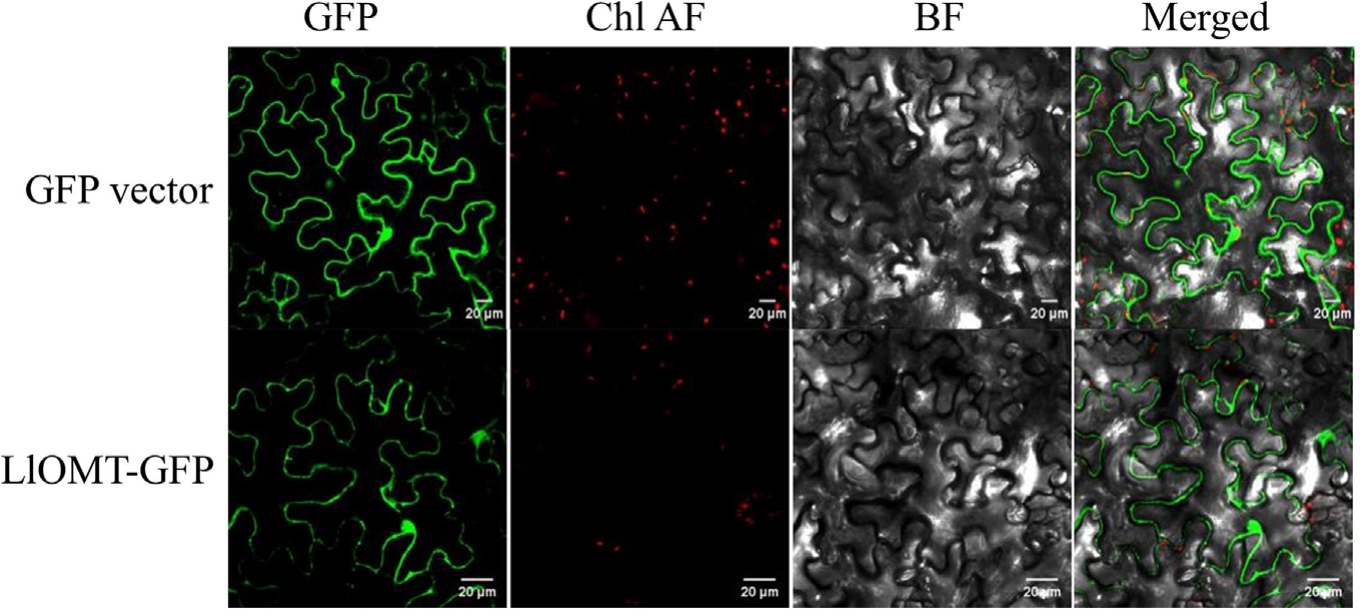
Subcellular localization of LlOMT-GFP fusion proteins transiently expressed in *N. benthamiana* leaves by *Agrobacterium* infiltration. Fluorescent signals of the GFP vector and LlOMT-GFP were observed by confocal microscopy. All bars = 20 μm. BF, bright field; Chl AF, chloroplast autofluorescence; GFP, green fluorescent protein; LlOMT, *Lycoris longituba* O-Methyltransferase (*N* = 3).

To elucidate the subcellular localization of LlOMT, translational fusions with enhanced GFP at the C-terminus that was driven by the CaMV 35S promoter were constructed for evaluating *Agrobacterium*-mediated infiltration of *N. benthamiana* leaves. Confocal microscopy revealed the occurrence of the LlOMT-GFP fluorescence signals in the cytoplasm of the mesophyll cells (**Figure 6**). The GPF vector fluorescent signal was also detected in the cytoplasm of the mesophyll cells (**Figure 6**).

### Expression of *LlOMT* and Gal Content in *L. longituba*

**Figure 7:**
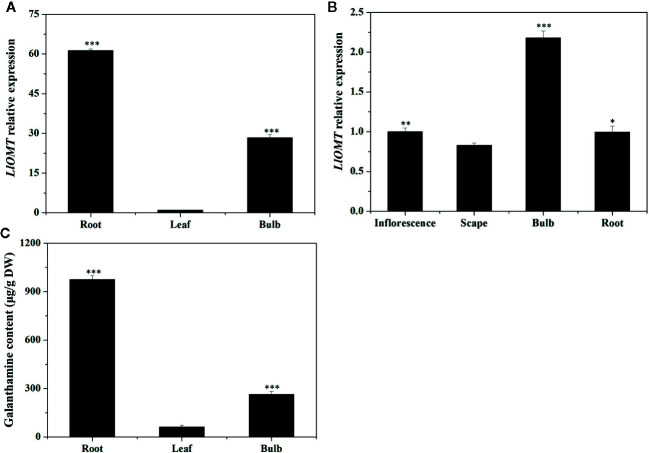
Expression of *LlOMT* and Gal content in *L. longituba*. LlOMT gene expression in the different tissues at the vegetative **(A)** and reproductive **(B)** phases; **(C)** Gal content in the different tissues at the vegetative phase. Gal, galanthamine; LlOMT, *Lycoris longituba* O-Methyltransferase (*N* = 3, *P < 0.05, **P < 0.01, ***P < 0.001 by Student’s *t*-test).

Gene expression of *LlOMT* in the different tissues at different stages was first examined by RT-qPCR. *LlOMT* expression was detected in all tissues of *L. longituba*, with high levels detected in R and B at the vegetative and reproductive stages (**Figure 7**). The Gal contents in the different tissues at the vegetative phase were then detected. The results showed that root had the highest Gal content (976.14 μg/g DW), which was significantly higher than other tissues. Gal content in bulb was 263.51 μg/g DW, and leaf tissue had the lowest Gal content (61.63 μg/g DW). The results indicated that Gal mainly accumulated in R, and the Gal content showed a similar pattern with *LlOMT* expression at the vegetative stage.

### LlOMT Enzyme Activity Assays

**Figure 8:**
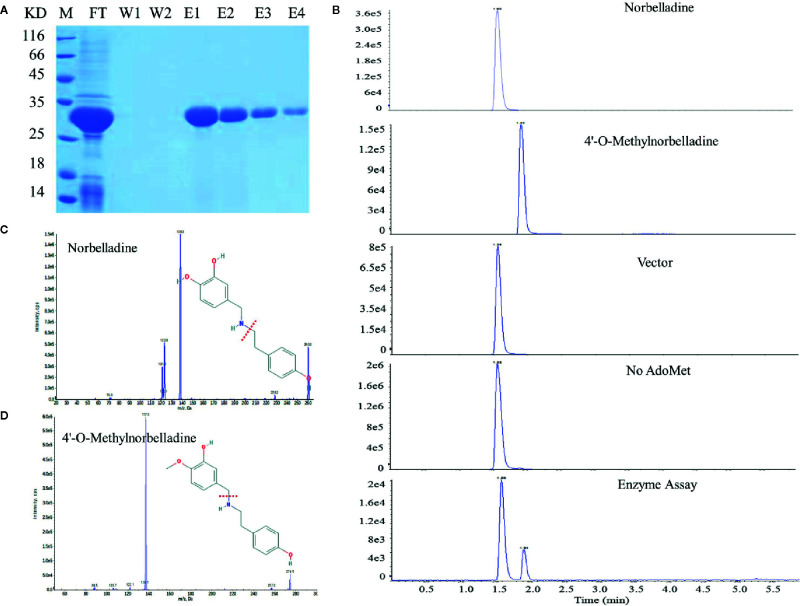
LlOMT enzyme activity assays of recombinant LlOMT. **(A)** SDS-PAGE gel image of LlOMT expression in *E. coli*. The recombinant His-tagged protein was purified from the culture lysates by Ni2+ affinity chromatography. FT, column flow-through sample; W1–W2, washing fractions. E1–E4, elution fractions. M, molecular weight marker. **(B)** LC-MS/MS analysis of the enzyme assay of LlOMT using norbelladine as a substrate. The reaction product 4’-O-methylnorbelladine was detected in an assay using recombinant LlOMT. The identity was confirmed by MS/MS fragmentation of norbelladine **(C)** and 4’-O-methylnorbelladine **(D)**. LlOMT, *Lycoris longituba* O-Methyltransferase; SDS-PAGE, sodium salt-polyacrylamide gel electrophoresis (*N* = 3).

NpN4OMT and LaOMT1 enzymes play key roles in the galanthamine biosynthesis pathway *via* the methylation of norbelladine to 4’-O-methylnorbelladine (Kilgore et al., 2014; Sun et al., 2018). Consequently, we also examined whether LlOMT exhibits catalytic activities. LlOMT was expressed as a *His*-tagged protein in *E*. *coli*. Following Ni^2+^ affinity purification, the recombinant protein was identified as a major band with the expected size of LIOMT (~29 kDa) after SDS-PAGE (**Figure 8A**). The purified *His*-tagged LlOMT protein was then tested with norbelladine as a substrate. The reaction assays indicated that 4’-O-methylnorbelladine was detected as a major peak in LC-MS/MS analysis (**Figures 8B–D**). The LlOMT-catalyzed O-methylation of norbelladine was found to follow the Michaelis and Menten kinetics pattern, the Km and Vmax values for the formation of 4′-O-methylnorbelladine was 317 ± 17 μM and 3.61 ± 0.14 μM min^−1^, respectively (**Supplementary Figure 9** and **Supplementary Table 8**).

**Figure 9:**
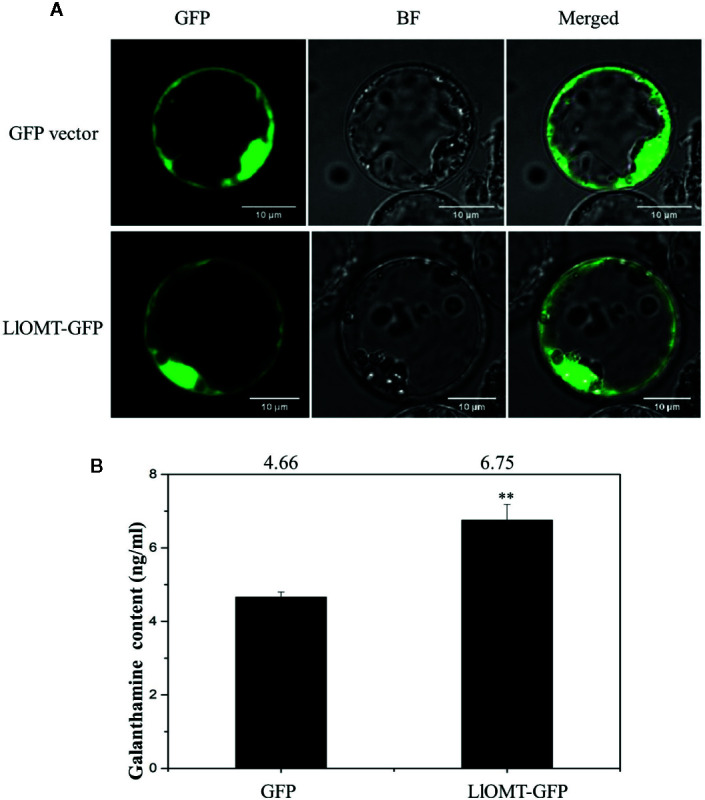
Overexpression of *LlOMT* in *L. longituba* protoplasts. Fluorescent signals of the GFP vector and LlOMT-GFP in the protoplast observed by confocal microscopy **(A)** and the Gal content in the protoplasts overexpressing the GFP vector and LlOMT-GFP **(B)**. Gal, galanthamine; GFP, green fluorescent protein; LlOMT, *Lycoris longituba* O-Methyltransferase (*N* = 3, **P < 0.01 by Student’s *t*-test).

To determine whether overexpressing LlOMT in *L. longituba* could increase Gal content, we transformed the LlOMT-GFP vector into *L. longituba* protoplasts using the GFP empty vector as a control. Confocal microscopy revealed the occurrence of the both fluorescence signals in the cytoplasm of the mesophyll cells (**Figure 9A**). Gal content analysis showed that overexpressing LlOMT could increase the Gal content by 1.45-fold compared with the control (**Figure 9B**).

## Discussion

The combination of sequencing technology and metabolite profiling has become a powerful tool for candidate genes identifications, and such integrated omics methods have been successfully used in non-model plants. For example, in mayapple, the six missing genes in etoposide aglycone biosynthetic pathways have been successfully characterized through transcriptome sequencing and metabolites analysis (Lau and Sattely, 2015); in *Narcissussp. aff. pseudonarcissus*, transcriptome sequencing and LC-MS/MS, NMR technology have been used to isolate *N4OMT* and *CYP96T1* genes (Kilgore et al., 2014; Kilgore et al., 2016).

In this study, transcriptome profiling and metabolite analysis of leaves, roots and bulbs of *L. longituba* were carried out, and 59 Gb of clean data was obtained with approximate 401 million clean reads and a total of 333,440 unigenes (average length 521 bp) were obtained by *de novo* assembly. Among them, 85,622 unigenes were successfully annotated. Recently, a transcriptome report of this specie was published by using 12 tepals samples at different stages as materials, and a large number of unigenes were also obtained (Yue et al., 2019). Although our study only use nine samples, we focused on diverse tissues including leaves, roots, and bulbs at vegetative growth stages. Our data is also the only transcriptome data including other tissues except the flower of *L. longituba* currently deposited in GenBank. The number of genes encoding different TF families varies among plant species and they often perform species-, tissue-, or developmental stage-specific function(s) (Smetanska, 2008; Yang et al., 2012; Wang et al., 2017). In this study, 38,347 unigenes encoding putative TFs were found, which could be divided into 58 TF families. Among them, the bHLH, MYB-related and MRKY families had been reported to regulate plant secondary metabolite biosynthesis (Shoji et al., 2010; Kazan and Manners, 2013; Phukan et al., 2016; Wang et al., 2017). These data sets will help us to further identify genes and metabolites in the secondary metabolic pathways. In particular, by comparing gene expression and metabolite accumulation, it is possible to generate a hypothesis for candidate genes involved in a particular biosynthetic pathway, as recently demonstrated by similar approaches (Geu-Flores et al., 2012; Yamazaki et al., 2013; Yonekura-Sakakibara et al., 2013; Enrique et al., 2017; Singh and Desgagné-Penix, 2017).

There are diverse groups of alkaloids in Amaryllidaceae plant family, and many biosynthetic enzymes are waiting to be discovered (Singh and Desgagné-Penix, 2014; He et al., 2015). Galanthamine (Gal) is a typical representative of amaryllidaceae alkaloids which is one of the main drugs for Alzheimer’s disease (Howes and Houghton, 2003; Repantis et al., 2010; Wang et al., 2010). Gal biosynthesis including several types of reactions, such as condensation, hydroxylation, oxidation, reduction, methylation, and phenol-phenol’ coupling (**Figure 4**; Singh and Desgagné-Penix, 2014). It has been reported that the initial steps of Gal biosynthetic pathway are also parts of phenylpropanoid metabolism (Hahlbrock and Scheel, 1989; Singh and Desgagné-Penix, 2014; Jaini et al., 2017). The catechol portion and non-catechol portion of Gal were derived from L-phenylalanine and L-tyrosine respectively (Barton and Kirby, 1962; Suhadolnik et al., 1962; Sandmeier et al., 1994). Phenylalanine ammonia lyase (PAL), cinnamate-4-hydroxylase (C4H) and coumarate-3-hydroxylase (C3H) were involved in the formation of 3,4-dihydroxybenzaldehyde from L-phenylalanine (Eichhorn et al.,1998). Tyrosine decarboxylase (TYDC) was involved in the formation of tyramine from L-tyrosine (Eichhorn et al.,1998; Takos and Rook, 2013). Recently, *LrPAL3* and *LrC4H* genes were cloned and characterized from *Lycoris radiata*, which may indicate the involvement of these genes in Gal biosynthesis (Li et al., 2018).

The core biosynthetic pathway of Gal required the formation of 3,4-dihydroxybenzaldehyde and tyramine, then the norbelladine and 4’-O-methylnorbelladine were produced through condensation, reduction, and methylation of the precursors (Kilgore et al., 2014; Kilgore et al., 2016). Recently, a transcriptome report of *L. radiata* was published by using six samples (two biological replicates) including leaves, roots and bulbs, and candidate genes involved in Gal biosynthesis were predicated without functional characterization (Park et al., 2019). In this work, seven genes which may be involved in the galanthamine metabolic pathway encoding tyrosine decarboxylase (TYDC), phenylalanine ammonia-lyase (PAL), cinnamate 4-hydroxylase (C4H), p-coumarate 3-hydroxylase (C3H), norbelladine synthase (NBS), norbelladine 4’-O-methyltransferase (OMT), and noroxomaritidine synthase (CYP96T1) were detected from the transcriptome data and validated by real-time quantitative PCR analysis (**Table 4**). The results of RT-qPCR showed that, these genes were differentially expressed in three tissues, and the highest expression level of *OMT* in roots may explained the highest amounts of Gal in this tissue. Similar relationships between *OMT* gene expression and Gal content could be observed in *N. pseudonarcissus* (Kilgore et al., 2014), *L. aurea* (Sun et al., 2018) and *L. radiata* (Park et al., 2019). Our results also found that Gal was mainly accumulated in root which was different from the results in *L. radiata*, *L. aurea*, and *N. pseudonarcissus*, among which high amounts of Gal were accumulated in bulb, ovary and bulb, respectively (Kilgore et al., 2014; Sun et al., 2018; Park et al., 2019). Such variations may due to species specificity, growth stages as well as environmental factors (Szakiel et al., 2011; Quan et al., 2012; Petruczynik et al., 2016). Our current study is likely to narrow down the genes involved in this pathway by homology, expression and metabolite analysis. The functional characterizations of these genes will be carried out in the future.

O-methyltransferase catalyzes a methylation reaction by transferring a methyl group from SAM to the acceptor’s hydroxyl group (Ibrahim et al., 1998). The identification of NpN4OMT and LaOMT1 showed that it is responsible for the methylation of norbelladine to 4’-O-methylnorbelladine which is the central intermediate in galanthamine biosynthesis (Kilgore et al., 2014; Singh and Desgagné-Penix, 2014; Sun et al., 2018; Li et al., 2019). In this study, *LlOMT* gene was identified from *L. longituba* in the proposed galanthamine biosynthetic pathway. Sequence analysis showed that LlOMT is a class I OMT and showed high protein similarity with NpN4OMT and LaOMT1 (**Supplementary Figure 8**). Similar with NpN4OMT and LaOMT1, LlOMT is localized in the cytoplasm (**Figure 6**), and biochemical analysis indicated that the recombinant LlOMT catalyzes norbelladine to generate 4’-O-methylnorbelladine (**Figure 8**). What’s more, the protoplast transformation result showed that the overexpression of *LlOMT* could increase the Gal content (**Figure 9**). Previous works on NpN4OMT1, LaOMT1 and LrOMT suggested different O-methylation preferences and substrate specificities of these proteins, LaOMT1 and LrOMT could form both meta- and para-O-methylated products, while NpN4OMT1 only generated para-O-methylated product (Kilgore et al., 2014; Sun et al., 2018; Li et al., 2019). These differences were speculated to be related with the amino acid differences in catalytic pocket (Li et al., 2019). In the future, substrate scope investigation should be carried out to better understanding the O-methylation type and preference of this protein. The higher expression of *LlOMT* and the larger concentration of Gal were observed in the root and bulb (**Figure 7**), suggesting a correlation between Gal accumulation and LlOMT gene expression. Our results indicate that LlOMT may play a role in galanthamine biosynthesis in *L. longituba*.

## Conclusions

Here, the genome size of *L. longituba* was determined and leaf, root, and bulb transcriptomes were generated from *L. longituba* for the first time. A very large dataset of transcripts and unigenes was generated, providing a rich genomic database from which to identify genes involved in secondary metabolite pathways. Notably, seven genes involved in the galanthamine biosynthesis pathway were identified in the transcriptomes. Moreover, the LlOMT gene was cloned and functionally characterized in *L. longituba* for the first time. These valuable gene candidates involved in the biosynthesis of galanthamine could be useful for producing larger quantities of bioactive compounds that are used in medical applications.

## Data Availability Statement

The datasets generated for this study can be found in the NCBI Sequence Reads Archive (SRA) with the accession number PRJNA590043.

## Author Contributions

QL, YZ, and YC designed the research. QL, JX, LY, and XZ performed the research. QL wrote the paper.

## Funding

This work was supported by the National Natural Science Foundation of China (No. 31801889), and by the Shanghai Rising-Star Program, China (No. 20QB1404100), and by the Shanghai plant seedling tissue culture professional technical service platform (No. 18DZ2291400).

## Conflict of Interest

The authors declare that the research was conducted in the absence of any commercial or financial relationships that could be construed as a potential conflict of interest.
