# Supplementary material for: Transcriptome Analysis of Different Tissues Reveals Key Genes Associated With Galanthamine Biosynthesis in Lycoris longituba
Source: Front Plant Sci. 2020 Sep 16;11:519752. doi: 10.3389/fpls.2020.519752 (PMC7525064; doi:10.3389/fpls.2020.519752)
Supplement: Supplementary file 3 [file Table_3.docx]

Supplementary Material

# Supplementary Tables

**Supplementary Table 1** | Primers used for RT-qPCR and cloning experiments.

| **Primer name** | **Sequences** | **Application** |
| --- | --- | --- |
| *PAL*-F | CAAAGTGCAGAGCAACATAATCAAG | qRT-PCR analysis |
| *PAL*-R | TTCACTGTGCTCTTCAAATTCTCC | qRT-PCR analysis |
| *C4H*-F | GTCAGAGGAATCTCGTAGTCGTGTC | qRT-PCR analysis |
| *C4H*-R | CTCACCGTACACTGTAAAGACCATG | qRT-PCR analysis |
| *C3H*-F | CAGGTGCTTCGCCGAGTGG | qRT-PCR analysis |
| *C3H*-R | CCTCACCTTCACGTAGTGGG | qRT-PCR analysis |
| *TYDC*-F | TTGGTGTCGGCGGATGAG | qRT-PCR analysis |
| *TYDC*-R | TGCTTCTCCTCCGTGAGG | qRT-PCR analysis |
| *NBS*-F | GAGTTGGAGGTTTCC TTGC | qRT-PCR analysis |
| *NBS*-R | CCACCATCACCTTCCTCG | qRT-PCR analysis |
| *OMT*-F | AAGCTTGTCAGGGTTGGAGG | qRT-PCR analysis |
| *OMT*-R | TACACTCCTCCTCTTCCGGA | qRT-PCR analysis |
| *CYP96T1*-F | TGCTATGGCGAGGATGAAGG | qRT-PCR analysis |
| *CYP96T1*-R | ACATGTCCCTTCACCATCTG | qRT-PCR analysis |
| *Actin*-F | CATCCCTCAGCACCTTCCAG | qRT-PCR analysis |
| *Actin*-R | CTGGGATGCAAAAACCGCC | qRT-PCR analysis |
| *OMT-GFP*-F | *cgatcgtctcacaac*ATGGGTGCTAGCCAAGATGAT | LlOMT-GFP vector |
| *OMT-GFP*-R | cagtcgtctcatacaATAAAGACGTCGGCAAATAGTC | LlOMT-GFP vector |
| *OMT-Protein* | cgccatatgATGGGTGCTAGCCAAGATGAT | OMT protein expression |
| *OMT-Protein* | ggcgaattcTCAATAAAGACGTCGGCAAATAGTC | OMT protein expression |

C3H, p-coumarate 3-hydroxylase; C4H, cinnamate 4-hydroxylase; CYP96T1, noroxomaritidine synthase; GFP, fluorescent protein; NBS, norbelladine synthase; OMT, norbelladine 4'-O-methyltransferase; PAL, phenylalanine ammonia-lyase; RT-qPCR, real-time quantitative polymerase chain reaction; TYDC, tyrosine decarboxylase.

**Supplementary Table 2** | Distribution of transcription factors (TF).

**Supplementary Table 3** | Gene Ontology (Go) annotation.

**Supplementary Table 4** | eggNOG classifications.

| **Category** | **Description** | **Number** | **Ratio (%)** |
| --- | --- | --- | --- |
| A | RNA processing and modification | 2,641 | 0.79 |
| B | Chromatin structure and dynamics | 1,453 | 0.44 |
| C | Energy production and conversion | 2,612 | 0.78 |
| D | Cell cycle control, cell division, chromosome partitioning | 1,087 | 0.33 |
| E | Amino acid transport and metabolism | 1,860 | 0.56 |
| F | Nucleotide transport and metabolism | 510 | 0.15 |
| G | Carbohydrate transport and metabolism | 3,045 | 0.91 |
| H | Coenzyme transport and metabolism | 717 | 0.22 |
| I | Lipid transport and metabolism | 1,676 | 0.5 |
| J | Translation, ribosomal structure and biogenesis | 4,780 | 1.43 |
| K | Transcription | 3,334 | 1 |
| L | Replication, recombination and repair | 7,969 | 2.39 |
| M | Cell wall/membrane/envelope biogenesis | 658 | 0.2 |
| N | Cell motility | 7 | 0 |
| O | Posttranslational modification, protein turnover, chaperones | 5,704 | 1.71 |
| P | Inorganic ion transport and metabolism | 1,685 | 0.51 |
| Q | Secondary metabolites biosynthesis, transport and catabolism | 2,187 | 0.66 |
| R | General function prediction only | 27,473 | 8.24 |
| S | Function unknown | 16,599 | 4.98 |
| T | Signal transduction mechanisms | 5,862 | 1.76 |
| U | Intracellular trafficking, secretion, and vesicular transport | 2,007 | 0.6 |
| V | Defense mechanisms | 449 | 0.13 |
| W | Extracellular structures | 117 | 0.04 |
| X | Undetermined | 0 | 0 |
| Y | Nuclear structure | 145 | 0.04 |
| Z | Cytoskeleton | 1,204 | 0.36 |

**Supplementary Table 5** | KEGG pathways.

| **Level1** | **Level2** | **Count** |
| --- | --- | --- |
| Metabolism | Overview | 279 |
| Metabolism | Carbohydrate metabolism | 403 |
| Metabolism | Energy metabolism | 294 |
| Metabolism | Lipid metabolism | 239 |
| Metabolism | Nucleotide metabolism | 150 |
| Metabolism | Amino acid metabolism | 266 |
| Metabolism | Metabolism of other amino acids | 110 |
| Metabolism | Glycan biosynthesis and metabolism | 93 |
| Metabolism | Metabolism of cofactors and vitamins | 176 |
| Metabolism | Metabolism of terpenoids and polyketides | 105 |
| Metabolism | Biosynthesis of other secondary metabolites | 126 |
| Metabolism | Xenobiotics biodegradation and metabolism | 51 |
| Metabolism | Enzyme families | 0 |
| Genetic information processing | Transcription | 216 |
| Genetic information processing | Translation | 516 |
| Genetic information processing | Folding, sorting and degradation | 372 |
| Genetic information processing | Replication and repair | 143 |
| Genetic information processing | RNA family | 0 |
| Environmental information processing | Membrane transport | 27 |
| Environmental information processing | Signal transduction | 398 |
| Environmental information processing | Signaling molecules and interaction | 1 |
| Cellular processes | Transport and catabolism | 256 |
| Cellular processes | Cell motility | 35 |
| Cellular processes | Cell growth and death | 181 |
| Cellular processes | Cellular community | 34 |
| Organismal systems | Immune system | 102 |
| Organismal systems | Endocrine system | 183 |
| Organismal systems | Circulatory system | 42 |
| Organismal systems | Digestive system | 56 |
| Organismal systems | Excretory system | 39 |
| Organismal systems | Nervous system | 116 |
| Organismal systems | Sensory system | 16 |
| Organismal systems | Development | 19 |
| Organismal systems | Aging | 76 |
| Organismal systems | Environmental adaptation | 0 |

KEGG, Kyoto encyclopedia of genes and genomes.

**Supplementary Table 6** | A[ssembly of RNA-seq data](http://www.baidu.com/link?url=W5z3DQzzmZoWQ39ax8iGvuwT-o0B8JbIoetNXzR7ObQNvEYHAP9D8nxV1VHbB7wl3rsTDU9oaMPLXNAOEz9RadEHR3p7M2jWWyiBbFSLJgW)

**Supplementary Table 7** | Expression[of RNA-seq data](http://www.baidu.com/link?url=W5z3DQzzmZoWQ39ax8iGvuwT-o0B8JbIoetNXzR7ObQNvEYHAP9D8nxV1VHbB7wl3rsTDU9oaMPLXNAOEz9RadEHR3p7M2jWWyiBbFSLJgW)

**Supplementary Table 8** | Kenetics parameters for LlOMT-catalyzed O-methylation (*N*=3).

| **Substrate/Product** | **Norbelladine/4’-O-methylnorbelladine** |
| --- | --- |
| *Km* (μM) | 317±17 |
| *Vmax* (μM min^-1^) | 3.61±0.14 |
| *Kcat* (min^-1^) | 0.361±0.014 |
| *Kcat/Km* (mM-1 min^-1^) | 1.139 |

# Supplementary Figures


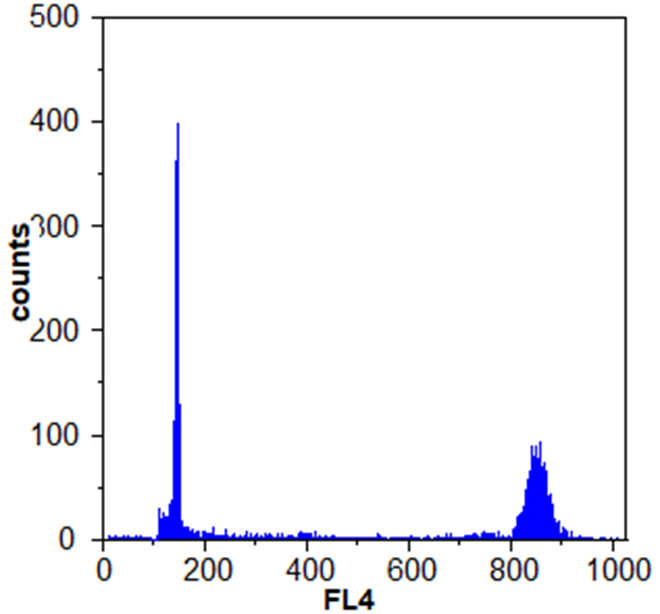


**Supplementary Figure 1.** Results of *L. longituba* and *H. vulgare* young leaf mixed samples from the flow cytometry analysis. P1: Peak of 2 C of *H. vulgare*, peak value is 151(CV=3.91); P2: Peak of 2 C of for *L. longituba*, peak value is 866 (CV=4.61).


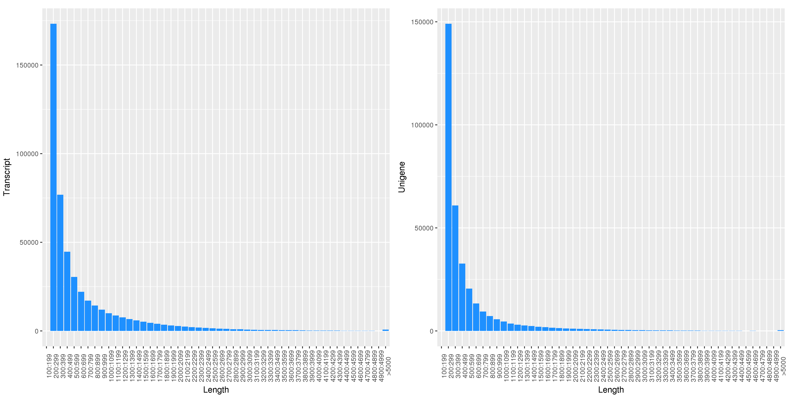


**Supplementary Figure 2.** Length distributions of the transcripts and unigenes.


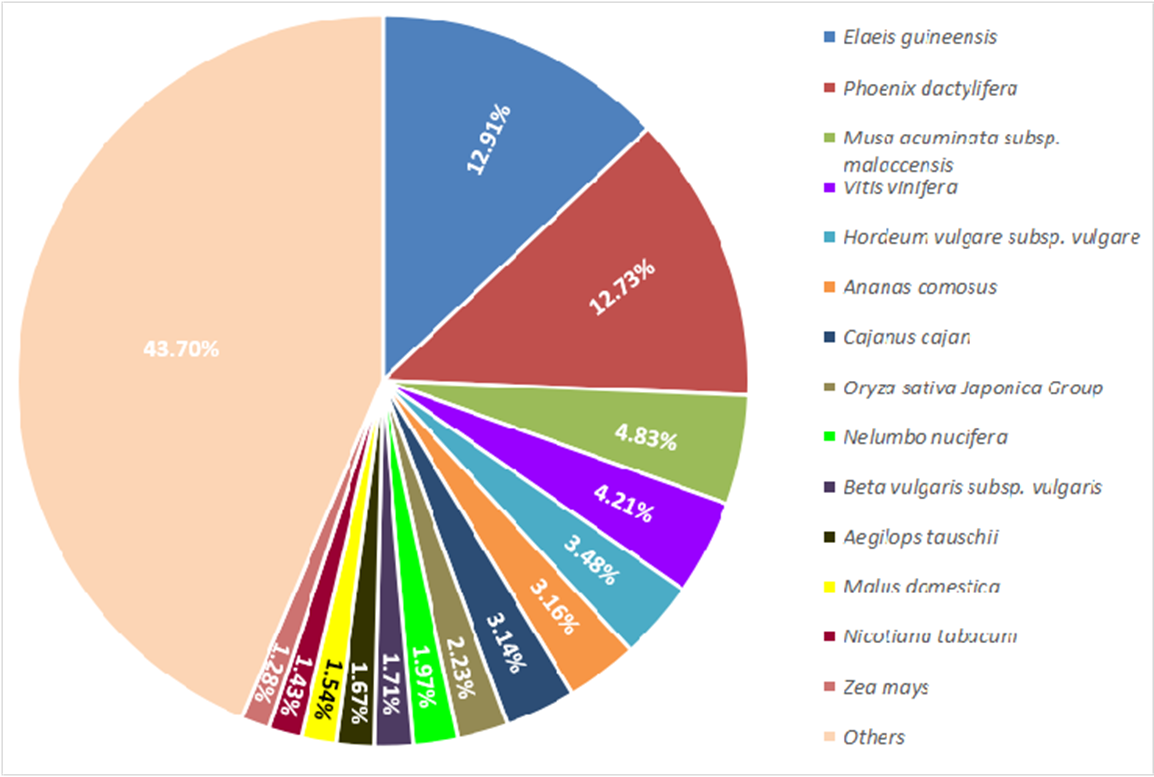


**Supplementary Figure 3.** Species homology analysis of the Nr annotation.


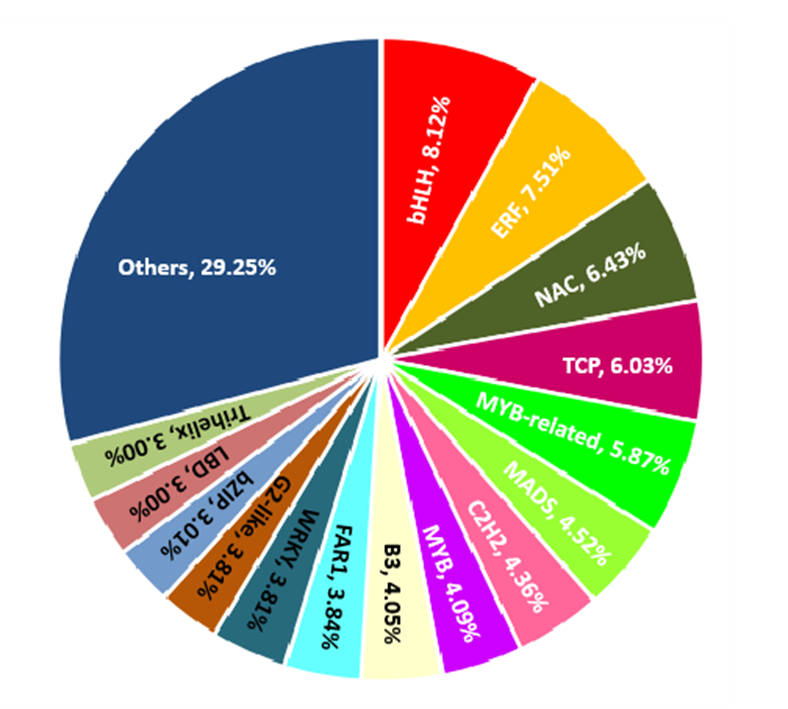


**Supplementary Figure 4.** Distribution of transcription factors (TF) families.


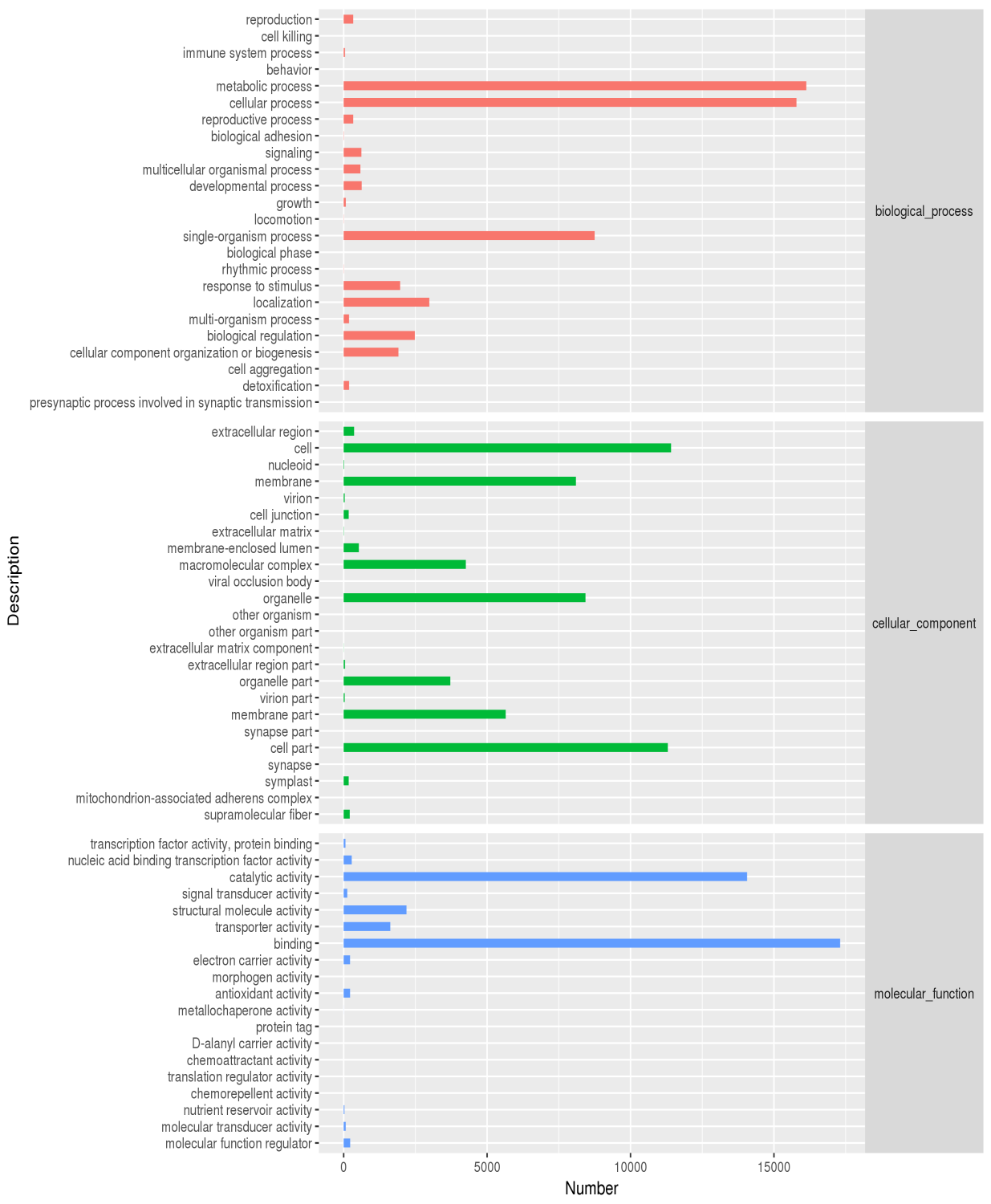


**Supplementary Figure 5.** Gene Ontology (GO) annotation.


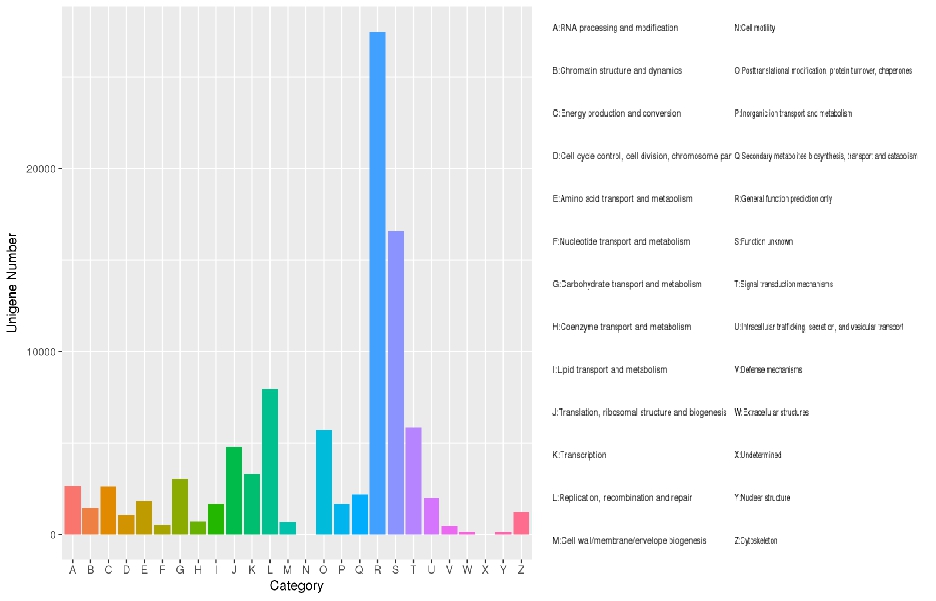


**Supplementary Figure 6**. eggNOG annotation.


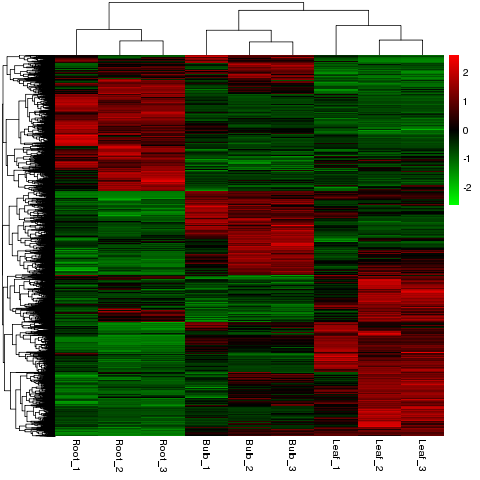


**Supplementary Figure 7**. Heatmap and cluster analysis of the differentially expressed genes (DEGs) of the different tissues.


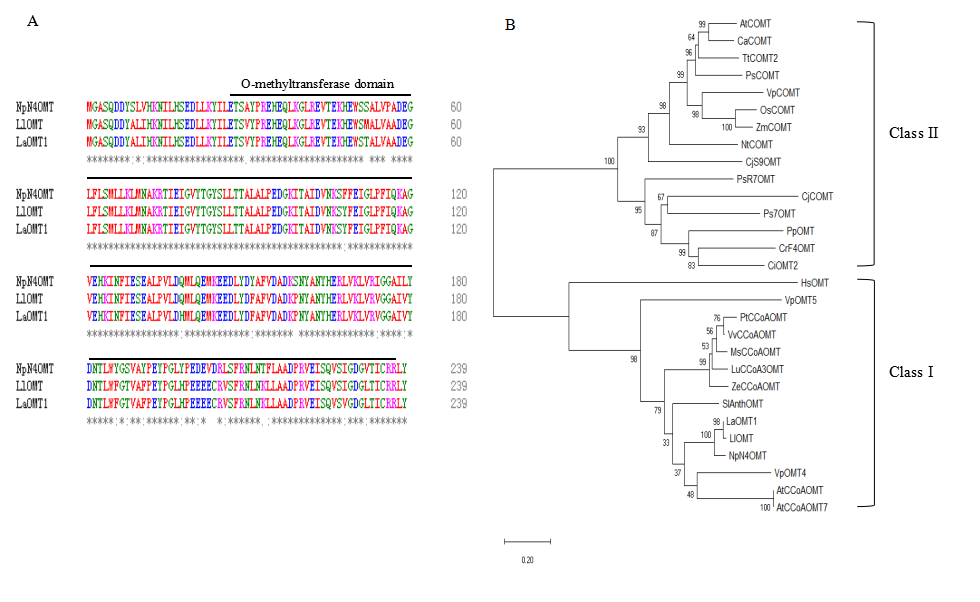


**Supplementary Figure 8**. Sequence analysis of LlOMT. (**A**) Protein sequence alignment of LlOMT1 with homologous proteins. (**B**) Phylogenetic analysis of LlOMT and other OMTs. Sequence alignment was performed with Clustal Omega, and the phylogenetic tree was constructed with MEGA version 5.2 using the neighbor-joining method with 1000 bootstrap replicates. Numbers at the nodes indicate the percentage bootstrap values. Scale bar = 0.2 amino acid substitutions.


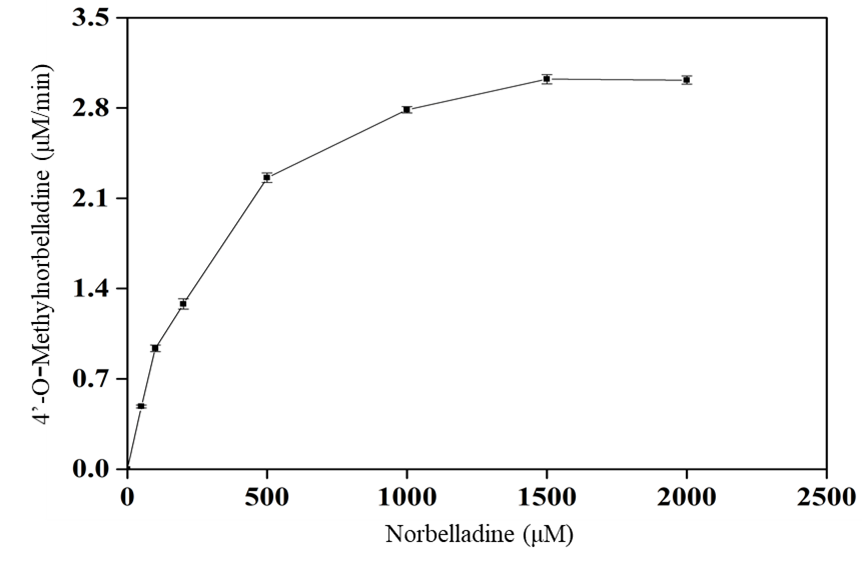


**Supplementary Figure 9**. Biochemical characterization of LlOMT. The effects of different ratios of norbelladine on the catalytic activity of LlOMT for enzymatic conversion of norbelladine into 4’-O-methylnorbelladine. The experimental values represent the means of three replicates ± standard deviation.
